# Chloroform Fatalities. IV

**Published:** 1895-09-28

**Authors:** Benjamin Ward Richardson


### Chloroform Fatalities.—IV.

By Sir Benjamin Ward Richardson, M.D., F.R.S.

In my last paper I discussed the influence of fear in regard to fatality from chlorofom and other anæsthetics, opening there a singular psychological question which I am not prepared at the present time to discuss further. I have no doubt in my own mind —indeed, I have expressed the same view for nearly a quarter of a century, that specially refined medicinal substances, especially those of a vaporous nature, and which, being in the blood, exert particular pressures, have their own individual influences, and change the condition of mind by the peculiar sympathy or physical state which they provoke or engender. I have shown that there are substances which, diffused through the body in minutest quantity, excite special dreams; and that even the body itself may generate, as if it were a chemical laboratory, certain substances that cause phenomena of a distinguishable character. But to enter minutely into this is too remote a point for our present practical purpose.

### Dilute Administration.

Setting aside these more refined considerations, of one thing I am quite sure, namely, that slow administration, or perhaps I had better say full administration, of a minute dose is the best and safest method. If I had been a direct follower of Dr. Snow in the work of practical anæsthetization; if I had called forth my best endeavours to make a profitable return from the anæsthetic practice he held, and which was placed in my hands, I could never have been popular as an administrator, because I objected to submit any of the confirmed *morituri* to the influence of the narcotic, and because, even when the healthy were submitted to me, I was thought to be too slow in producing insensibility to pain, and would not, as it was said, "push on the chloroform" with the celerity that operators desired. It is true I have never lost a case, and on the rarest occasions have seen even danger; but the general opinion seemed to be that I did not proceed with sufficient celerity. I think at this date that the caution which I learned from Snow, and perhaps exaggerated from him, is becoming more in favour owing to the excessive number of deaths that have occurred from an opposite line of practice. I have just had before me a record of cases and phenomena by Dr. R. W. Carter, of Weymouth, who has ascertained, after careful administration, that from 0·32 up to 3 per cent. of chloroform in the respired air answers every purpose of chloroform anæsthesia. He has also paid special attention to the scientific administration of ether, and found that by the same method of minute administration, from 22 to 60 minims of ether per minute answers every purpose of ether anæsthesia. The results in his hands from his notes of forty administrations stand before me in illustration.

The smallest quantity of chloroform expended per minute, on the average, during the narcotisation induced by Dr. Carter in using chloroform is 1·4 minims, while the maximum he gives is 10 minims. The smallest quantity of ether is 22 minims, the largest 120 minims. He has demonstrated the superiority of chloroform over ether, a superiority which seems to depend upon the fact that the comparatively large quantity of ether entirely fails to induce anæsthesia if it be inhaled with air in the same manner as chloroform always should be inhaled; that is to say, the action of ether must be assisted by making the patient inhale his own expired air over and over again in combination with the ether, by which a certain degree of asphyxia must be induced, as well as any particular anæsthesia which the ether may, on its part, produce. From some cases of anæsthesia by ether I find that Dr. Carter has succeeded in holding a patient free from the pain of operation of excision of the breast for 60½ minutes, using during the whole time only 2 ounces 7 drachms of ether—an average of 22·8 minims per minute. In another case he succeeded for 12 minutes, at the rate of 1½ ounce, or 60 minims per minute. In a third case he kept up the administration for 28 minutes on 2 ounces of ether, or at the rate of 25·2 minims per minute. In a further case, in an administration of seven minutes, he employed 6 drachms of ether, or 51 minims per minute.

The advantages which are claimed upon these results of ethereal administration as against fatal results, or signs of such results, are: colour, which is usually natural; breathing without stertor or irregularity; good condition of the pulse; no perspiration; reduction of the phenomena of sickness; and, rapid and good recovery.

I reserve for a final paper the question whether ether or other ethereal forms are likely to be as useful as bodies of the chloroform type and less dangerous; but I conclude this paper by urging that before we come to a definite conclusion on the subject of fatality from the chloroform we require to be quite sure whether a modification in the administration of chloroform, by which as small a quantity as possible of chloroform can be used, may or may not render chloroform as safe as ether. This can only be effected by unanimity in observation. So long as a number of administrators go on from day to day, each using a narcotic according to his own fancy—or, as he would probably say himself, his own experience—the great question at stake can never be properly solved. In an ordinary experiment carried on in the laboratory for the solution of a question in which the matter of human life is not at all concerned, it would be a subject of joke if every man at work proceeded on his own solution. It would be much the same in a game of chess. What we want, then, in administration of chloroform, in order to learn how to reduce fatalities to the smallest figure, is for all administrators to try and prove one general and apparently most acceptable plan, and for them then to meet, say, after twelve months, and compare their researches with those which have gone before, on the irregular system, in which quantities and modes of administration have represented the most varied changes. It goes with reason to believe that a system by which it was determined to administer at the same temperature of air, and with the smallest possible amount of chloroform, would yield the safest and best results; but we must still be prepared for certain failures amongst the *morituri*, and must isolate the effects of fear from the fatal effects of the anæsthetic itself.

